# An Introduction to Practical Chemistry, Including Analysis

**Published:** 1866-12

**Authors:** 


AN INTRODUCTION TO PRACTICAL CHEMISTRY, INCLUDING ANALYSIS. By JOHN E. BOWMAN, F.C.S., late Professor of Practical Chemistry in King's College, London, etc., etc. Edited by CHARLES L. BLOXAM, F.C.S., Professor of Practical Chemistry in King's College, London, etc., etc. With 107 Illustrations. Fourth American, from the fifth revised London edition. Philadelphia: HENRY C. LEA. 1866.

The former editions of this very convenient and valuable little work, are already familiar to the profession. The editor has made some additions to the present edition; and it will be found one of the best guides for the student of practical chemistry, and a very convenient manual for reference by the profession generally. It is a small volume of 351 pages.

For sale by W. B. KEEN & Co., 148 Lake St., Chicago.

